# Supplementary material for: An instrument to assess HIV-related knowledge and adjustment to HIV+ status, and their association with anti-retroviral adherence
Source: PLoS One. 2020 Jun 22;15(6):e0227722. doi: 10.1371/journal.pone.0227722 (PMC7307754; doi:10.1371/journal.pone.0227722)
Supplement: Attachment — Submitted filename: HealthLitARV.docx [file pone.0227722.s001.docx]

The relationship between literacy and HIV adherence is well known. The strength of this study is that it incorporates facets of meaning, identity, and biomedical knowledge in creating a comprehensive assessment of knowledge among adults living with HIV. The paper has several strengths, including providing detailed information about the pilot testing and incorporating the feedback of an advisory board. They also conducted cognitive interviewing to improve the scale.

My main areas to improve the paper relates to the methodology and analysis.

General comments

Referring to people living with HIV: Authors used a variety of labels HIV+; people with HIV, living with HIV. Since "people living with HIV" is the more acceptable term, they can consider using it consistently.

Methods

- The methods indicate that two modalities were used for data collection: online and through a research assistant. Comments are
  a. Were there any differences in the results by the two methods
  b. What is the interclass correlation (ICC) between those two modalities?
  c. What proportion completed the survey online and in-person

What was the inclusion and exclusion criteria for the study?

Analysis

- Need for cognition scale: Since this scale is central to the results of the paper, the authors are encouraged to report relevant reliability and validity literature of the scale in the text. For example, what is the internal consistency and validity of the scale?
- In addition, they should also give some examples of items in the Need for cognition scale, explain how it is scored, and interpreted.
- Their health literacy scale seems to have five sections (as described in the methods). It is unclear whether when the factor analysis was done was all of the items across the multiple domains included. For example, only two factors were reported from the Factor Analysis, and the items do not include the knowledge quizzes. So it is unclear if their health literacy scale assessed only the personal meaning of living with HIV or included the knowledge quizzes? If this is the case, why didn't they include the knowledge items, since they wanted to create a comprehensive assessment of health literacy?
- Relatedly, in Table 1, it may be helpful to have sub-categories, so that readers are aware of what items relate to what sections/domains of the scale.
- The analysis is very scant, and more details are needed. First, what was the analytical process for the factor analysis? What rotations did they use? What indices of fitness did they use to arrive at the two factors?
- In addition, in the results section, under the "need for cognition," they share the results of two separate analyses (page 11, lines 7 and 8). In one analysis, they controlled for quiz score, and another, for cognition. These analyses and the rationale behind them are not detailed in the methods section.
- The authors argue that this scale is more comprehensive. They encourage its use within clinical settings. What is missing, however, is the validity of the scale. Language about validity is missing. For example, I can see the relationship between literacy and ARV adherence as construct validity. They can also report on the discriminant validity of the scale, using items they know are not related to literacy.
- They should indicate the level of significance used and the statistical package for analysis.
- There is no justification for the sample size; thus, it is unclear whether the lack of association, for example, between biomedical knowledge and ARV adherence was due to a small sample size.

Results

- The authors did not provide any data on the socio-demographic and medical history of the participants. Since participant backgrounds may influence their knowledge and self-identity, it will be helpful here for them to report these results. For example, what is the level of education of the participants? How long have they been living with HIV? What are the age categories? What was the level of internalized stigma within the population (was any data collected here) since stigma is also an essential part of patient experience and identity
- The authors do not report the overall Cronbach's alpha of the scale, nor the sub-scales, although the individual alphas are reported.
- One page 9, line 17, they reported the mean of the ARV treatment items and commented that there was a considerable variance. They should report the variance.

Discussion

- The authors do not report any limitations of the study.
- Can they compare the reliability of their literacy measure to that of other studies?
- Comments on validity?
