# Supplementary material for: An instrument to assess HIV-related knowledge and adjustment to HIV+ status, and their association with anti-retroviral adherence
Source: PLoS One. 2020 Jun 22;15(6):e0227722. doi: 10.1371/journal.pone.0227722 (PMC7307754; doi:10.1371/journal.pone.0227722)
Supplement: Attachment — Submitted filename: Response_to_reviewers.docx [file pone.0227722.s002.docx]

Response to Reviewers

*Reviewers’ comments are in italics.*

Reviewer 1

*The relationship between literacy and HIV adherence is well known. The strength of this study is that it incorporates facets of meaning, identity, and biomedical knowledge in creating a comprehensive assessment of knowledge among adults living with HIV. The paper has several strengths, including providing detailed information about the pilot testing and incorporating the feedback of an advisory board. They also conducted cognitive interviewing to improve the scale.*

Thank you. We hope we have shed more light on the relationship between literacy and HIV adherence.

*My main areas to improve the paper relates to the methodology and analysis.*

*General comments*

*Referring to people living with HIV: Authors used a variety of labels HIV+; people with HIV, living with HIV. Since "people living with HIV" is the more acceptable term, they can consider using it consistently.*

We have consistently referred to PLWH after first spelling it out.

*Methods*

- *The methods indicate that two modalities were used for data collection: online and through a research assistant. Comments are*
  a. *Were there any differences in the results by the two methods*
  b. *What is the interclass correlation (ICC) between those two modalities?*
  c. *What proportion completed the survey online and in-person*

Only 20 people completed the questionnaire on-line. Given that small number, it is probably not informative to check the differences.

*What was the inclusion and exclusion criteria for the study?*

The only requirements were that people identified themselves as HIV positive and were able to complete the questionnaire in English. We have added this statement to the methods (line 8).

*Analysis*

- *Need for cognition scale: Since this scale is central to the results of the paper, the authors are encouraged to report relevant reliability and validity literature of the scale in the text. For example, what is the internal consistency and validity of the scale?*

Chronbach’s alpha for the NFC has been reported to be .89 or .90 depending on age group. Test-retest reliability has been reported to be r=.76. Higher levels of NfC are correlated with higher levels of cognitive abilities, estimated IQ, and education. (Soubelet and Salthouse, 2017). We have added this information. (page 5)

- *In addition, they should also give some examples of items in the Need for cognition scale, explain how it is scored, and interpreted.*

The scale is provided as Table 4, with the direction of scoring indicated.

- *Their health literacy scale seems to have five sections (as described in the methods). It is unclear whether when the factor analysis was done was all of the items across the multiple domains included. For example, only two factors were reported from the Factor Analysis, and the items do not include the knowledge quizzes. So it is unclear if their health literacy scale assessed only the personal meaning of living with HIV or included the knowledge quizzes? If this is the case, why didn't they include the knowledge items, since they wanted to create a comprehensive assessment of health literacy?*

As we explain in the results, three of the sections were not correlated with ARV adherence so we deleted them in order to create a parsimonious instrument. We did not factor analyze the knowledge quizzes because the answers are either true or false. The tables contain all of the items that we retained and show where the factor analysis was done. We realize this was not clearly explained in the methods, we have revised accordingly.

- *Relatedly, in Table 1, it may be helpful to have sub-categories, so that readers are aware of what items relate to what sections/domains of the scale.*

We aren’t sure we understand this comment. We factor analyzed this scale and found a two-factor solution, which we show.

- *The analysis is very scant, and more details are needed. First, what was the analytical process for the factor analysis? What rotations did they use? What indices of fitness did they use to arrive at the two factors?*

We used varimax orthogonal rotation, which simplifies the interpretation of the factors by maximizing the variances of the squared loadings for each factor. The interpretation of the two factors is intuitively obvious, and the first factor is associated with ARV adherence.

- *In addition, in the results section, under the "need for cognition," they share the results of two separate analyses (page 11, lines 7 and 8). In one analysis, they controlled for quiz score, and another, for cognition. These analyses and the rationale behind them are not detailed in the methods section.*

Again, we have revised the methods to explain this more clearly. (Page 7)

- The authors argue that this scale is more comprehensive. They encourage its use within clinical settings. What is missing, however, is the validity of the scale. Language about validity is missing. For example, I can see the relationship between literacy and ARV adherence as construct validity. They can also report on the discriminant validity of the scale, using items they know are not related to literacy.

There are actually four scales in our instrument, as shown in the four tables. Each of them is associated with self-reported adherence, which constitutes construct validity. We believe that it is responses to individual items, particularly on the knowledge quizzes, that is most likely predictive, rather than the overall score, i.e. a particular misconception underlies non-adherence. The usefulness in a clinical setting is then to diagnose the reasons for non-adherence in particular individuals, which may be addressable.

- *They should indicate the level of significance used and the statistical package for analysis.*

0.05, SAS 9.4 We have added this information to the methods section.

- There is no justification for the sample size; thus, it is unclear whether the lack of association, for example, between biomedical knowledge and ARV adherence was due to a small sample size.

Given that we had no basis for assessing prior probability, we could not do a power analysis in advance. The sample size was the largest we could obtain within our budget, and it is typical of pilot tests of instruments. There was not even a trend for association between the items we deleted and adherence. If there is an association it is very small. This observation is consistent with our formative qualitative research, as referenced. (Laws, et al, 2015)

Results

- *The authors did not provide any data on the socio-demographic and medical history of the participants. Since participant backgrounds may influence their knowledge and self-identity, it will be helpful here for them to report these results. For example, what is the level of education of the participants? How long have they been living with HIV? What are the age categories? What was the level of internalized stigma within the population (was any data collected here) since stigma is also an essential part of patient experience and identity*

We provide this information on page 8 under “Participant characteristics, with the exception of internalized stigma which we did not assess as such. However, items Q2-1j and Q2_1r in table 1 are direct references to stigma.

- *The authors do not report the overall Cronbach's alpha of the scale, nor the sub-scales, although the individual alphas are reported.*

Overall alpha of the scale shown in table 2 is .84. We have added this information.

- *One page 9, line 17, they reported the mean of the ARV treatment items and commented that there was a considerable variance. They should report the variance.*

The sd was 2.69. We have added this information.

Discussion

- *The authors do not report any limitations of the study.*

We have added a statement about limitations.

- Can they compare the reliability of their literacy measure to that of other studies?

We are unaware of the existence of any measures of HIV-related knowledge (other than questionnaires about transmissibility) for which reliability has been assessed.

- Comments on validity?

We do show the association of our measures with self-reported adherence, which speaks to validity.
